# Beyond interferon side effects: What residual barriers exist to DAA hepatitis C treatment for people who inject drugs?

**DOI:** 10.1371/journal.pone.0207226

**Published:** 2018-11-30

**Authors:** Annie Madden, Max Hopwood, Joanne Neale, Carla Treloar

**Affiliations:** 1 Centre for Social Research in Health, UNSW Sydney, Sydney, Australia; 2 National Addiction Centre, Kings College London, London, United Kingdom; Burnet Institute, AUSTRALIA

## Abstract

Recent advances in the efficacy and tolerability of hepatitis C treatments and the introduction of a universal access scheme for the new Direct Acting Antiviral (DAA) therapies in March 2016, has resulted in a rapid increase in the uptake of hepatitis C treatment in Australia. Despite these positive developments, recent data suggest a plateauing of treatment numbers, indicating that more work may need to be done to identify and address ongoing barriers to hepatitis C treatment access and uptake. This paper aims to contribute to our understanding of the ongoing barriers to DAA therapies, with a focus on people who inject drugs. The paper draws on participant interview data from a qualitative research study based on a participatory research design that included a peer researcher with direct experience of both hepatitis C DAA treatment and injecting drug use at all stages of the research process. The study’s findings show that residual barriers to DAA treatment exist at personal, provider and system levels and include poor venous access, DAA treatments not considered ‘core-business’ by opioid substitution treatment (OST) providers, and patients having to manage multiple health and social priorities that interfere with keeping medical appointments such as childcare and poor access to transport services. Further, efforts to increase access to and uptake of DAA hepatitis C treatment over time will require a focus on reducing stigma and discrimination towards people who inject drugs as this remains as a major barrier to care for many people.

## Introduction

With the relatively recent advent of direct acting antiviral (DAA) therapies, hepatitis C (HCV) treatment has taken a significant step forward in efficacy and tolerability [1]. DAA treatments show higher rates of cure, of shorter duration and with significantly lower side effect profiles than previous longstanding interferon-containing regimens [2, 3]. These advances have given rise to the possibility of the “elimination of HCV as a public health problem”, with the World Health Organisation (WHO) setting the goal of an 90 per cent reduction in HCV incidence at the global level by 2030 [4].

The Australian Government has also committed to the WHO Global Elimination Targets and, in March 2016, commenced the provision of a universal access scheme for HCV treatment that provides affordable access to the DAA regimens via government subsidy [5]. Access to the DAA therapies is further enhanced by the introduction of non-invasive pre-treatment diagnostics (such as transient elastography) [6], the implementation of GP and nurse-led models of care [7, 8] and the availability of government funded retreatment for people who have undertaken HCV treatment unsuccessfully with the previous interferon-based therapies.

These developments have resulted in a rapid increase in the uptake of HCV treatment with the DAA therapies by people with chronic HCV infection in Australia. Recent treatment data show that between March 2016 and March 2017 approximately 38,500 of Australia's estimated 230,000 people with HCV had been treated—this represents almost 17 per cent of Australia's total HCV population [9]. As approximately 80% of people living with HCV in Australia are estimated to have acquired the infection via injecting drug use [10], there is need to understand the issues relevant to this group.

Despite these impressive numbers, recent data are showing a plateauing in treatment uptake [11] which highlights that the goal of eliminating HCV as a public health concern will require further work to identify and remove ongoing barriers to treatment and care. There is a growing literature which explores the barriers to HCV treatment uptake remain following the introduction of the more tolerable and effective DAA therapies [12]. This paper aims to contribute to our understanding of these residual barriers to care in the era of DAA treatments among people who inject drugs. Consistent with the literature in the area, this paper assessed barriers to HCV care at the patient, provider and system levels [13] to identify where action is required now if we wish to maintain or enhance the uptake of HCV treatment into the future.

## Methods

### Study design

The participatory design of this study included the involvement of a peer researcher with direct experience of both injecting drug use and HCV treatment with DAAs in all aspects of the knowledge production process. This aspect of the design was a key part of the effort of this study to support self-determination of affected communities [14, 15]. The involvement of a peer researcher (AM) was also useful for creating a ‘safe space’ for the exploration of the subjective experiences of people who inject drugs (a highly marginalised community) in an era of rapidly expanding biomedical responses to HCV treatment.

### Data collection

In-depth interviews were conducted with 24 participants recruited through community-based networks of people who injected drugs in Melbourne, Victoria, Australia in collaboration with a community partner organisation, Harm Reduction Victoria. A poster advertising the study with information on eligibility criteria and how to contact the peer researcher was placed at the Needle & Syringe Program at the reception of Harm Reduction Victoria and the peer workers at the organisation were also encouraged to refer people directly to the peer researcher. Particpants were also encouraged to refer people from their peer networks to the peer researcher for assessment about inclusion in the study. Inclusion criteria were being diagnosed with chronic HCV infection, aged 18 years or over, living in Australia and having a recent history of injecting drug use (past six months). Six participants were recruited across each of four groups to represent a range of engagement with DAA treatment to enable examination of remaining barriers: (1) people who had refused or deferred treatment for HCV with DAAs; (2) people who were actively thinking about, planning and/or about to commence treatment for HCV with DAAs; (3) people currently undertaking treatment for HCV with DAAs; and, (4) people who had recently completed treatment for HCV with DAAs.

Interviews were conducted between December 2016 and March 2017, were of 20–30 minutes’ duration and all were conducted by the peer researcher. Participants were asked to discuss: a biographical snapshot including experience of diagnosis; knowledge and expectations of HCV treatment; current health and wellbeing; future life following treatment and basic demographic information. Each participant received AUD $20. Approval for this study was provided by the Human Research Ethics Committee of UNSW, Sydney.

The interviews were audio-recorded, transcribed verbatim and edited to remove any information that may have identified participants. An inductive analytical approach was used to shed light on the ways in which individuals understood themselves, their health and decisions about it, the health care system (including the clinical encounter or other activities in the health system) and their social worlds relevant to HCV treatment [16]. These experiences were thematically categorized to identify barriers to accessing HCV treatment or care. The themes were then further analysed using a patient/provider/system structure to identify and assess ongoing barriers to HCV treatment uptake and outcomes such as knowledge and perceptions of people living with HCV, service provider attitudes and values and system level policies and infrastructure. This approach acknowledges that impaired access to treatment often involves multiple barriers to care, present at various points across the system, and that addressing these barriers requires action across patient/provider/system levels [13]. We recognize that the use of “patient” is not strictly correct for some participants (as they had not taken up DAA treatment). We use “patient” in this sense to examine issues that affect the personal experience.

## Findings

The sample included nine women and 15 men with an age range of 28–63 years. In Australia, surveillance studies of people who inject drugs typically report a gender mix of approximately two-thirds men [17] and monitoring of DAA uptake indicates that two-thirds of people treated were men [11], which is reflected in the sample for this study. Two participants identified as Aboriginal Australians. Three participants reported unstable housing and eight were living in public housing. Most (n = 15) received government benefits as their main source of income and eight reported full-time or part-time employment. Nine participants reported completing high school education to Year 12 or above. Table 1 includes details of the sample in each of the four participant groups.

**Table 1:** Demographic characteristics of participants by group (total n = 6 participants per group.

	Group 1	Group 2	Group 3	Group 4	Total (n = 24)
Women	3	3	2	1	9
Men	3	3	4	5	15
Age range (years)	32–64	28–54	32–58	33–63	28–64
Aboriginal and/or Torres Strait Islander	1	1	0	0	2
Unstable housing	1	1	1	0	3
Public housing	1	3	2	2	8
Government benefits as main source of income	3	4^a^	4	4^a^	15
Regular Employment (f/t or p/t)	3	2	2	1	8
Completed high school education^b^	1	3	2	3	9

^a^one person receiving government support for full-time study

^b^ Completed Year 12 or above in the Australian education system.

### Personal barriers to DAA treatment

#### Being asymptomatic

Participants reported a range of post HCV-diagnosis and pre-treatment experiences and emotional responses, including being ‘shocked’ (male, group 4, 33 years); ‘scared’ (male, group 2, 38 years); ‘nervous and upset’ (male, group 1, 49 years) and ‘I thought I was going to die’ (female, group 1, 50 years). Following diagnosis, most people acknowledged that being ‘asymptomatic’ (male, group 2, 50 years) made them disinclined to monitor their infection post-diagnosis and/or to seek treatment. Participants said that they ‘often forgot they had HCV’ (female, group 3, 42 years) due to the lack of obvious symptoms. Participants also reported their limited understandings of the impact of HCV on liver health and explained that this information was not provided at diagnosis or other moments in their care. No, it was just ‘really oh hep C, is that something to do with hepatitis?’ So, a lot of naivety and not really understanding the potential consequences and how it could affect you in later life. So, this was back in 1991, we’re now in 2016. I never really did anything about it I never worried about it. I never seem to have any symptoms that I’m aware of. (female, group 3, 58 years)

#### Finding the ‘right time’ for DAA treatment

Participants spoke about ‘deferring treatment’ for a long time for numerous reasons including ‘not being sick’ (male, group 3, 34 years), ‘having other responsibilities and lifestyle issues to manage’ (female, group 1, 50 years), and ‘being pregnant’ (female, group 2, 41 years). Others spoke about having constantly postponed treatment, waiting for the ‘perfect moment’ when everything would ‘align’ to maximise the likelihood of a good treatment outcome (male, group 2, 50 years). Sometimes participants spoke about there being ‘no point’ (female, group 3, 42 years) to commencing HCV treatment while they were still injecting drugs as they were afraid of the risk of reinfection and the possibility of being denied access to future treatment.

Deciding when to have treatment was also influenced by provider- and system-level factors. Participants stated that they had often been advised by health providers ‘to wait for the new treatments’ (male, group 4, 33 years) or that they were ‘waiting for the new treatments to be available and affordable’ (male, group 4, 53 years). Others were confused about their eligibility for treatment while currently injecting, often due to the negative attitudes of health professionals.

I think some of the doctors think, ‘you know I’m prepared to prescribe it for the odd client who’s known to me, but I don’t want those people in my waiting room. I certainly don’t want to open myself up to those people’ and so even though you know … it was no longer technically a barrier to treatment, there was so many specialists who just would not … who just simply didn’t think that current users were appropriate cases for treatment, or eligible. (female, group 1, 64 years)

#### Poor vein health

Poor vein health was also identified as a key barrier to DAA treatment and this issue represented a significant cross-over between individual- and provider-level barriers. Veins can be damaged after years of injecting drug use and efforts by inexperienced or poorly trained staff to access blood can lead to further damage, pain and frustration and make future blood testing more difficult. In this study, some participants required several appointments so that sufficient blood could be drawn for testing.

The big barrier for me is no one can get blood out of me, I’ve just got no veins and I mean this has been a problem for the last 10 or so years and … it’s just impossible for me to give blood and I guess that’s what partly sort of sharpened my perception of just how under-rated and under-valued the whole issue of testing has been within the new treatment discussions. It really hasn’t been identified as a key component of treatment. I mean we are talking about a community of people with veins that have been used often for many, many years so they’re going to be compromised at best and damaged in many cases and just that there’s no alternative. Like it’s not that there is even one specialist clinic where it’s known there are really clever phlebotomists on site… (female, group 1, 64 years)

Sometimes, the problem of blood testing led to a renegotiation of expertise between clinicians and participants, as some participants were better at accessing their own blood than their doctors and health workers. Phlebotomists reportedly had tried to access groin arterial blood, which was described by one participant as ‘very painful’ (male, group 3, 34 years), requiring this participant to take control of the situation because he was able to access blood from his wrist. Another participant said that he would not let health professionals near his ‘good vein’ (male, group 4, 56 years) but instead preferred to endure pain from blood draws during DAA treatment (even preferring them to take blood from his neck) to protect his healthy vein. Participants reported that in these situations it was less painful and quicker for them to draw their own blood than to have health workers attempt the procedure.

… a lot of them are incompetent and they just jab away and wreck my veins, so it’s just quicker too and less painful if I can do it and then they get relieved and I’m relieved and everybody’s relieved and they’ve got their blood. (female, group 3, 42 years)

In addition to expanding opportunities for people who inject drugs to be able to draw their own venous samples, some participants were aware of testing protocols such as reflex testing whereby two blood draws are taken at one time [18] and new technologies such as point of care RNA and finger-prick capillary blood testing [19] as potential strategies to minimise the need for multiple blood draws and/or significantly reduce the need for traditional venipuncture among people with compromised veins. For some participants however, there was still concern about how these new technologies would operate in the real world.

… I’ve read about perhaps dry blood spot PCR’s in the future however, you know there’s still going to be other stuff that needs to be done. I don’t think it’s going to be as easy as, ‘oh we’re 12 months down the track, now we can just do a PCR’. (female, group 1, 50 years)

### Provider barriers to DAA treatment

#### Gaps in continuity of care

When participants were ready to undertake treatment, finding a health professional to prescribe treatment was sometimes not straightforward, despite a policy of universal access to DAA treatments in Australia. Specifically, two participants talked about their general practitioner (GP) or opioid substitution treatment (OST) prescriber initially refusing to initiate their DAA treatment regimen on the basis that they didn’t see HCV as their core business, and they thought patients should access HCV treatment through a specialist service. One participant was able to convince her OST prescriber to provide DAA treatment after initial refusal. This is a resolution for this instance, but also suggested that other people with HCV who may not have such resources or resilience may have accepted the initial decision.

… because she’s a methadone doctor I figured she’d be all over it, but that was prior to the GP’s being allowed to do it and then the GP’s were allowed to do it and so I hit my doctor up right away and honestly didn’t expect her to go, ‘no I’m not going to do it’. Fucking drove me nuts. I got furious at her. (female, group 3, 42 years)

The difficulties participants had in accessing and undergoing interferon-based HCV treatment provided in tertiary services, contributed significantly to people’s lack of engagement with their infection and to a perceived reluctance of health services to provide follow-up care. Those who did engage in regular monitoring said they mainly did so to ‘stay in contact’ (female, group 3, 58 years) and to be ‘on the spot’ (male, group 4, 53) when DAA treatments became available. While a few participants spoke about their GP or OST prescriber regularly ‘checking in’ with them following their initial diagnosis (male, group 3, 32 years), most participants reported few significant conversations with health providers about accessing regular monitoring and/or accessing treatment. In these cases, participants described the post-diagnosis waiting period as frustrating, adding that they did not feel like their health providers were taking their HCV infection seriously.

I wasn’t offered any support at all. It was just ‘this is what you’ve been diagnosed with, we can give you treatment now, but it won’t be the best for you, just wait, there’s new treatment coming, we can’t tell you exactly when its coming, but it will be much better and more beneficial for you.’ (male, group 4, 33 years)

I just remember being told ‘you’ve got hep C’ and it was just like ‘oh yeah whatever’. It was like ‘you’ve got a cold’ or ‘you’ve got the flu’ that’s it. (female, group 3, 58 years)

### System-level barriers to DAA treatment

#### Managing multiple health and social priorities

For some participants, particularly those on opioid pharmacotherapies and/or those managing ongoing dependent drug use, barriers to accessing HCV treatment were complex and related to multiple linked or layered issues and services that were not equipped to operate in a coordinated or integrated fashion. For example, participants spoke about the difficulty of getting ancillary support such as help with transport or childcare services that would allow them to attend HCV treatment services, and they emphasized the frustration they experienced in their attempts to co-ordinate their care. One participant explained how living with multiple chronic conditions impeded access to HCV treatment when key support services failed to respond to the broader circumstances in people’s lives. The experience of this participant was also bound up with a concern about how she would be perceived by health providers when her presentation was influenced by a range of medications.

For example, last year when I had my family service worker, that’s the reason I didn’t get the treatment done, because I was like being sick from my methadone, so I couldn’t travel so far unless I really took a lot of medications and I didn’t want to get up to the hospital like that…. They might have refused to dose me or something and so I needed to get there and they wouldn’t do it. They refused to do it … their job was to get me to appointments, because I had panic attacks around a lot of people, in queues, what does my psychologist call it, ‘social anxiety’ and so I did need help and so I got the scans and everything done ready to go start my treatment and no one would bring me. I was sick as hell and so three times I rescheduled the appointment and I thought, ‘oh my god, this is just hopeless’ and so unless I could find someone who is going to help me and get me to where I’ve got to go, there’s no point, so just wait. And so I thought, I’m going to find a GP in the northern suburbs that I can get to and not rely on anybody else. (female, group 2, 41 years)

#### Finding supportive and non-judgmental care

Participants discussed their experiences of stigma and discrimination in health care settings. Examples included emergency doctors telling participants they ‘were sick of dealing with you junkies’ (male, group 3, 34 years) and pharmacists making participants wait for lengthy periods before being served, speaking to them in a poor manner and generally not observing common courtesies to customers with injecting histories. Often, discussion of stigma centred around participants’ sense that clinicians viewed them as inappropriate clients to be receiving DAA treatment, which resulted in the internalization of these messages.

I still hide it, no matter what… I just won’t do it…um, and yeah that’s from fear of judgment I’d say but I don’t think it would have really mattered. It’s more from me… it’s the stigma within as much as the stigma without. (male, group 4, 56 years)

There was a level of suspicion among some participants about the health system, the confidentiality of clients’ health records, and the level of surveillance that may occur when a client accesses treatment. Similarly, participants reported their perception that clinicians generally view people who inject drugs as not caring about their health and that reinfection is a likely outcome of HCV treatment, making DAA therapy an expensive ‘waste of time’ (male, group 3, 34 years) for this population. One participant commented that poor attitudes toward people who inject drugs are reflected in the low number of GPs that have ‘embraced’ (female, group 1, 64 years) the opportunity to prescribe DAAs. To overcome systems-level and social barriers to DAA treatment, participants explained that people who inject drugs need information about where they can access services and support to have DAA therapy without experiencing stigma and discrimination.

Participants also reported that there is still a high level of concern and ignorance in the general community about HCV and how it is transmitted, which they believed underpinned a lot of the stigma they encountered. Clients’ families were often described as misunderstanding HCV, injecting and drug dependence, believing affected people were ‘weak’ and had no ‘willpower’ to address their health conditions: ‘if you were strong enough you could do it’ (female, group 2, 41 years). Similarly, participants stated that media depictions of HCV reduced the likelihood of affected people disclosing their infection, which in some families had led to needless and damaging suspicion.

My mum treats me like I’ve got AIDS … like the other day, she bought a juice and I wanted to have a taste of it, she said, “no because I might catch your Hep C” … She’s so paranoid. (female, group 2, 54 years)

I still don’t tell anyone, I don’t disclose, never disclose. Even at the dentist I leave it blank. … Discrimination. Not for me, for my kids. You know it’s for them, I don’t want them being judged because of my mistakes. (female, group 2, 41 years)

## Discussion

Australia enjoys world-leading access to HCV DAA treatment and has committed to achieve elimination of HCV in advance of the global targets. However, these goals will not be achieved without attention to the barriers which remain for those who have yet to undertake DAA treatment, particularly for people who inject drugs. These findings show that significant barriers remain at patient, provider and system levels and that these barriers can reinforce or compound each other and that stigma associated with drug use and HCV is entwined with many of these barriers [20]. Hence, we should examine remaining barriers not in silos but seeing the interconnections between experiences over time and settings.

The journey to DAA treatment passes through many moments of interaction between patient and health providers. Some participants in this study described receiving no information or support for understanding the impact of HCV on the liver at diagnosis or in subsequent interactions. In past research, a lack of knowledge has been identified as a barrier to DAA treatment uptake including among people who inject drugs engaged with services [21]. Engaging with people living with HCV will require addressing gaps in knowledge or misunderstandings that at times may have existed for years or decades. This includes more recent changes, such as misperceptions that re-treatment will be denied people who acquire a new infection after treatment success. While this can be seen as a “patient” barrier, it requires a response that mobilizes providers and systems to address this gap and connect people with accessible care in ways that make sense and are authentic to them [22].

Finding DAA prescribers was a further barrier identified in these results. In Australia, there are growing numbers of DAA prescriptions being written outside of tertiary hospitals; general practitioners and medical staff in alcohol and other drug services represent growing segments of the DAA prescriber profile [23]. However, how to find health professionals who prescribe DAA treatment will be especially difficult for those with limited material resources to travel distances to other sites or limited social, psychological and health resources to endure a number of attempts to connect with care [24]. Intermediary services are required, which can support people with multiple complex needs to ensure those who are most vulnerable to these system-level barriers are not further excluded.

Despite growing awareness of new developments such as point of care testing [19], these findings suggest that people who inject drugs perceive that poor vein health was not well understood by health providers as a significant barrier to DAA treatment. Encouraging people who are concerned about their veins into testing will require efforts at both provider level (explaining and demonstrating their skill) and system level (developing and implementing new technologies). Some innovations such as reflex testing (two blood draws at one time to enable antibody testing and follow-up PCR testing) have demonstrated success in achieving completion of testing and in supporting efforts to maximize engagement with the cascade of care [18]. The importance of this innovation for people with vein problems has achieved less attention [25].

Efforts to remove DAA treatment barriers also need to take account of pervasive stigma and discrimination related to HCV and injecting drug use. Again, action in this area requires attention across patient/provider/system levels. People with HCV and who inject drugs may have received numerous direct and indirect messages over their lives that they are less worthy citizens [20, 26] and not legitimate candidates for HCV treatment [27]. As we understand stigma as a cause of health inequalities [28], efforts to overcome the barriers to DAA treatment should emphasise the rights of people who inject drugs to access supportive and non-stigmatising care and to be provided with information on where and how people can register their complaints, should this not be the case.

This study was a qualitative study of a small number of individuals who self-reported HCV infection and recent injecting drug use. These data do not identify specific barriers in locations, or throughout service areas, nor do the data inform ways to address these barriers at the local level. These data were collected during the early phase of implementation of universal access to DAA therapies in Australia. It may be that work has since occurred to address some barriers, for example, by identifying strategies to better support GPs and OST prescribers to work with clients with HCV.

The exciting opportunity to eliminate HCV as a public health challenge has galvanized action in many countries, such as Australia. As with any intervention, there remains the issue of how to minimise the barriers and maximize the inclusion of people with fewer resources [29]. This study’s findings show that a sophisticated, multifaceted approach is required to increase treatment uptake over the longer term, and to improve the experience of DAA treatment. Such approaches must look across actors (patient, provider, system), across time (acknowledging and addressing deficits in previous care experiences), and across settings (community, primary care and tertiary care). Efforts to increase DAA treatment uptake must reduce stigma and discrimination as a central precept to any effective response.
